# Interoceptive Impairments Do Not Lie at the Heart of Autism or Alexithymia

**DOI:** 10.1037/abn0000370

**Published:** 2018-08

**Authors:** Toby M. Nicholson, David M. Williams, Catherine Grainger, Julia F. Christensen, Beatriz Calvo-Merino, Sebastian B. Gaigg

**Affiliations:** 1 School of Psychology, Keynes College, University of Kent; 2 Department of Psychology, University of Stirling; 3 Department of Psychology, City, University of London

**Keywords:** autism, alexithymia, interoception, heartbeat tracking, self-awareness

## Abstract

Quattrocki and Friston (2014) argued that abnormalities in interoception—the process of representing one’s internal physiological states—could lie at the heart of autism, because of the critical role interoception plays in the ontogeny of social-affective processes. This proposal drew criticism from proponents of the *alexithymia hypothesis*, who argue that social-affective and underlying interoceptive impairments are not a feature of autism per se, but of alexithymia (a condition characterized by difficulties describing and identifying one’s own emotions), which commonly co-occurs with autism. Despite the importance of this debate for our understanding of autism spectrum disorder (ASD), and of the role of interoceptive impairments in psychopathology, more generally, direct empirical evidence is scarce and inconsistent. Experiment 1 examined in a sample of 137 neurotypical (NT) individuals the association among autistic traits, alexithymia, and interoceptive accuracy (IA) on a standard heartbeat-tracking measure of IA. In Experiment 2, IA was assessed in 46 adults with ASD (27 of whom had clinically significant alexithymia) and 48 NT adults. Experiment 1 confirmed strong associations between autistic traits and alexithymia, but yielded no evidence to suggest that either was associated with interoceptive difficulties. Similarly, Experiment 2 provided no evidence for interoceptive impairments in autistic adults, irrespective of any co-occurring alexithymia. Bayesian analyses consistently supported the null hypothesis. The observations pose a significant challenge to notions that interoceptive impairments constitute a core feature of either ASD or alexithymia, at least as far as the direct perception of interoceptive signals is concerned.

## General Scientific Summary

This article suggests that impairments in interoception—the process of representing one’s internal physiological states—do not lie at the heart of either autism or alexithymia.

Interoception refers to the representation of one’s internal physiological states, such as breathing, hunger, thirst, and heart rate (Craig, 2003). Recently, studies have linked interoceptive accuracy (IA; i.e., the extent to which one can detect interoceptive signals accurately) to a number of important psychological functions, such as emotion processing (Barrett, Quigley, Bliss-Moreau, & Aronson, 2004; Shah, Catmur, & Bird, 2016; Zaki, Davis, & Ochsner, 2012), empathy (Fukushima, Terasawa, & Umeda, 2011), theory of mind (Demers & Koven, 2015; Shah, Catmur, & Bird, 2017), and self-awareness (Seth, 2013). These associations support theories that suggest cognition is “embodied” (Gallese & Sinigaglia, 2011; Glenberg, 2010; Goldman & de Vignemont, 2009), and imply that interoception impairments might play a critical role in psychological disorders. Indeed, impairments in interoception have been implicated recently in increasingly prominent theories of one developmental disorder, in particular: namely, autism spectrum disorder (ASD; Quattrocki & Friston, 2014).

ASD is a neurodevelopmental disorder diagnosed on the basis of behavioral impairments in social-communication and behavioral flexibility (American Psychiatric Association [APA], 2013). At the cognitive level, difficulties with theory of mind (Yirmiya, Erel, Shaked, & Solomonica-Levi, 1998), emotion processing (Gaigg, 2012), and psychological aspects of self-awareness (Williams, 2010) are well-established among people with ASD. Moreover, approximately 50% of autistic individuals have clinically significant levels of alexithymia (Berthoz & Hill, 2005; Hill, Berthoz, & Frith, 2004; Joukamaa et al., 2007; Milosavljevic et al., 2016)—a condition characterized by difficulties with representing, understanding, and describing one’s own emotional states (Taylor, 1984). This collection of impairments led Quattrocki and Friston (2014) to suggest that impairments in interoception, due to a dysfunctional oxytocin system, may be the root cause of autism. Oxytocin is a hormone and neurotransmitter that plays a significant role in regulating social-affiliative behaviors. Quattrocki and Friston (2014) argue that a dysfunctional oxytocin system could lead to abnormalities in the production and modulation of interoceptive signals, and their integration with exteroceptive information about the environment. As a result, autistic children develop impoverished internal models of emotional states (i.e., alexithymia) and the self, more generally, while external social-emotional signals hold less salience and therefore lead to the specifically characteristic social-affective impairments of the disorder. A crucial prediction that follows from this view is that IA should be associated with the number of ASD traits manifested by a person and also be reliably impaired among people with a diagnosis of ASD (Hypothesis 1).

In contrast, others have argued that self-awareness difficulties in ASD should be restricted to awareness of one’s own cognitive and emotional states (i.e., the *psychological* self), leaving basic *detection* of one’s own physiological states (i.e., the *physical* self) essentially unimpaired (Lind, 2010; Uddin, 2011; Williams, 2010). This view is derived from observations of a preserved sense of agency (Cascio, Foss-Feig, Burnette, Heacock, & Cosby, 2012; Paton, Hohwy, & Enticott, 2012; Schauder, Mash, Bryant, & Cascio, 2015) and preserved self-recognition (Dawson & McKissick, 1984; Ferrari & Matthews, 1983; Lind & Bowler, 2009) in ASD. This account, therefore, leads to the prediction that IA should not be associated with ASD traits or impaired in people with a full diagnosis of ASD (Hypothesis 2).

Distinct from the two views above is the more recent idea that many of the social-affective difficulties experienced by people with ASD are, in fact, the result of co-occurring alexithymia, rather than the result of ASD itself (Bird & Cook, 2013; Bird et al., 2010; Cook, Brewer, Shah, & Bird, 2013; Oakley, Brewer, Bird, & Catmur, 2016). According to this alexithymia hypothesis, difficulties with emotion processing and empathy are only apparent in people with ASD who also have high levels of alexithymia (or comorbid alexithymia). Most important, unlike Quattrocki and Friston (2014) who view interoceptive abnormalities as central to the etiology of ASD, the alexithymia hypothesis considers interoceptive abnormalities central to the etiology of alexithymia (Brewer, Happé, Cook, & Bird, 2015; Hatfield, Brown, Giummarra, & Lenggenhager, 2017; Herbert, Herbert, & Pollatos, 2011; Shah, Hall, Catmur, & Bird, 2016). This leads to the prediction that IA should be associated with the level of alexithymia, rather than the number of ASD traits, manifested by a person and that it should be significantly impaired only among people with ASD who manifest clinically significant levels of alexithymia (Hypothesis 3).

Although distinguishing the three competing predictions above is of central importance to our understanding of the defining features of ASD, only four studies have assessed IA in this population and the results are highly inconclusive. In line with *H*_1_, Garfinkel and colleagues (Garfinkel, Tiley, et al., 2016) found *reduced* accuracy among 20 adults with ASD on a classic heartbeat-tracking task that required participants to keep count of their heartbeats without physically taking their own pulse. Likewise, Palser, Fotopoulou, Pellicano, and Kilner (2018) found reduced IA on the heartbeat-tracking task in 30 autistic children. In contrast, Schauder et al. (2015) observed no impairments on the same heartbeat-tracking task in a group of 21 autistic children. In keeping with *H*_2_, this study furthermore found that heartbeat tracking accuracy was correlated with a test of bodily awareness on which children with autism were also unimpaired, thus confirming a putative link between preserved interoception and a preserved awareness of the physical self in ASD. Finally, Gaigg, Cornell, and Bird (2016), using a physiological arousal tracking paradigm, and Shah, Catmur, et al. (2016), using the traditional heartbeat-tracking task, obtained partial evidence for *H*_3_ by showing that IA was unimpaired in adults with ASD who were matched to a comparison group on self-reported levels of alexithymia. This group matching ensured that levels of alexithymia were experimentally controlled when examining IA, leading the authors to suggest that interoceptive impairments *would* have been observed in ASD if groups had *not* been matched on alexithymia and that differences in findings across other studies (e.g., Garfinkel, Tiley, et al., 2016; Schauder et al., 2015) likely reflect differences across ASD samples in the prevalence of alexithymia.

The evidence concerning interoception in ASD is not only difficult to interpret because of the inconsistencies across studies, but also because three of the five studies essentially argue for the null hypothesis (i.e., no difference between ASD and comparison groups) on the basis of very small samples. Moreover, in the studies of Gaigg et al. (2016) and Shah, Catmur, et al. (2016), the group-matching strategy meant that the majority of individuals scored in the subclinical range for alexithymia, rendering the ASD groups nonrepresentative of the wider autism spectrum where an estimated 50% of individuals manifest alexithymia (Berthoz & Hill, 2005). In other words, the central claim of the alexithymia hypothesis—that interoceptive impairments would be observed in individuals with ASD who have clinically significant levels of alexithymia—remains untested. To test this prediction and contrast it effectively with the alternative predictions outlined above, it is necessary to compare IA among individuals with ASD who report clinically significant alexithymia (ASD-high alexithymia) with IA among matched individuals with ASD who report subclinical levels of alexithymia (ASD-low alexithymia). If *H*_1_ is correct, regardless of alexithymia level, individuals with ASD should show impaired interoception relative to an NT comparison group. In contrast, if *H*_2_ is correct, individuals with ASD should show unimpaired interoception, regardless of alexithymia level. Finally, if *H*_3_ is correct, then only ASD-high alexithymia individuals should manifest diminished IA, with the ASD-low alexithymia individuals demonstrating equivalent IA to the neurotypical (NT) group. In Experiment 2, we tested these predictions among 46 individuals with ASD and 48 NT participants. First, however, Experiment 1 examined the associations predicted by *H*_1_ and *H*_3_ among IA, alexithymia, ASD traits, and theory of mind in 137 NT individuals.

## Experiment 1

### Method

#### Participants

One hundred thirty-seven students (114 female) from the University of Kent took part in Experiment 1. The average age of participants was 19.73 years (*SD* = 2.98; range = 18–23 years). No participant had a history of ASD, according to self-report. All participants gave informed consent and received course credit in partial fulfilment of their degree, for taking part in the study. The experiment was approved by the School of Psychology Research Ethics Committee, University of Kent (Ethics ID: 201714870662234338).

#### Materials and procedures

Interoception was measured using a standard heartbeat-tracking task (Schandry, 1981). In a quiet room, participants were asked to close their eyes and, without taking their pulse, silently count their heartbeat during four different time intervals (25, 35, 45, and 100 s), which were presented in a randomized order. An auditory tone signaled the beginning and end of each time interval. A pulse oximeter (Contec Systems CMS-50D+; Qinhuangdao, China) attached to participants’ index finger measured their actual heart rate. IA was calculated as 1 – (recorded number of heartbeats − counted number of heartbeats)/[(recorded heartbeats + counted number of heartbeats)/2] by Garfinkel, Seth, Barrett, Suzuki, and Critchley (2015). This provided a value between −1 and 1 for each time interval, with more positive values indicating better IA.^1^ It should be noted that the heartbeat-tracking task has come under recent scrutiny, with some claiming that the task is not always/necessarily a valid measure of interoception (see Brener & Ring, 2016, for a review). We consider this concern further in the Discussion section, below. Here, we note only that the heartbeat-tracking task is (for better or worse) by far the most widely used measure of interoception in the literature, in part because of ease of administration and in part because monitoring one’s own heartbeat is fundamental to emotional experience. The task has good test–retest reliability (Mussgay, Klinkenberg, & Rüddel, 1999), is sensitive to individual differences (Christensen, Gaigg, & Calvo-Merino, 2018; Dunn et al., 2010; Garfinkel et al., 2015) and is mediated by brain regions that are involved in awareness of one’s physiological states (Critchley, Wiens, Rotshtein, Öhman, & Dolan, 2004; Pollatos, Schandry, Auer, & Kaufmann, 2007). Nevertheless, to address some of the concerns that exist in relation to this task, we also present some additional analyses in Supplement 1 (see Point 4 of the online supplemental materials, in particular).

The 50-item Autism-Spectrum Quotient (AQ; Baron-Cohen, Wheelwright, Skinner, Martin, & Clubley, 2001) was administered as a self-report measure of autistic traits, and the 20-item Toronto Alexithymia Scale (TAS-20; Bagby, Parker, & Taylor, 1994) as a self-report measure of alexithymia. Each questionnaire requires participants to indicate to what extent a series of statements applies to them (e.g., AQ: “I find social situations easy”; TAS-20: “I have feelings that I can’t quite identify”), with scores on the AQ ranging from 0–50 (scores >26 are suggestive of a possible diagnosis of autism) and scores on the TAS-20 ranging from 20–100 (scores >60 indicate clinically significant alexithymia). In addition, participants completed the Reading the Mind in the Eyes (RMIE) task (Baron-Cohen, Wheelwright, Hill, Raste, & Plumb, 2001). This widely recognized measure of emotional theory of mind requires participants to decide which of four mental state terms (mostly emotional in nature) best describes the feelings conveyed by the eye region of faces as portrayed in 36 unique photographs. Scores range from 0–36, with higher scores indicating better emotional theory of mind. We included the RMIE task specifically because it differentiates groups of ASD participants from groups of NT participants and because it taps those emotional aspects of theory of mind that are thought to be related to alexithymia (Oakley et al., 2016). Given that recent studies have shown an association between IA and emotional, but not nonemotional, aspects of mind reading (Shah et al., 2017), we included the RMIE to investigate the relation between emotional theory of mind and interoception.

#### Statistical power and analysis

An a priori power calculation using G*Power3 (Faul, Erdfelder, Buchner, & Lang, 2009) revealed that, to detect an association between IA and TAS score of *r* = −.37 (as found by Herbert et al., 2011, and Shah, Catmur, et al., 2016) on 80% of occasions using two-tailed tests, 55 participants are required. A larger sample was recruited here to allow for the cross-validation of the findings, by randomly splitting the total sample into two subsamples (*n* = 68 and 69, respectively), each with sufficient power (>.90) to detect a reliable association between TAS-20 score and IA. Bayesian analyses were also conducted (using JASP 0.8.1; JASP Team, 2016) to provide a more graded interpretation of the data than is possible using *p* values or effect sizes alone (e.g., Dienes, 2014; Rouder, Speckman, Sun, Morey, & Iverson, 2009). Bayes factors (BF_10_) >3 provide firm evidence for the alternative hypothesis (with values >10, >30, and >100 providing strong/very strong/decisive evidence) and values under 1 provide evidence for the null (with values <0.33 providing firm evidence; Jeffreys, 1961).

### Results

Among the entire sample, the 137 participants scored a mean of 25.82 (*SD* = 3.97) on the RMIE task, 16.99 (*SD* = 6.66) on the AQ, and 50.82 (*SD* = 10.29) on the TAS. The mean IA score on the heartbeat detection task was .50 (*SD* = .27). Crucially, there was no significant association between IA and TAS total score, *r* = .008, *p* = .92, BF_10_ = 0.11, or between IA and any of the other variables: (AQ total score: *r* = −.11, *p* = .22, BF_10_ = 0.22; RMIE: *r* = .03, *p* = .73, BF_10_ = 0.11). TAS total score was, however, associated significantly with both AQ total score, *r* = .42, *p* < .001, BF_10_ > 100, and RMIE, *r* = −.24, *p* = .005, BF_10_ = 5.16, whereas AQ total score and RMIE were not significantly associated in the current sample, *r* = .11, *p* = .21, BF_10_ = 0.24 (and note that the association between TAS and RMIE remained significant after controlling for AQ, *r*_*p*_ = −.21, *p* = .01).^2^ Finally, Fisher’s *Z* tests revealed that the IA × TAS correlation was significantly different from that reported by Herbert et al. (2011), *Z* = 3.35, *p* < .001, and Shah, Catmur, et al. (2016), *Z* = 2.03, *p* = .04. For ease of reference, this pattern of correlations is set out in Table 1 along with those for Experiment 2.[Table-anchor tbl1]

Although IA and TAS were not significantly associated, it may be that IA would nonetheless be significantly impaired among individuals with significant levels of alexithymia. To investigate this, the current sample was divided according to scores on the TAS. Those with scores above the cutoff were assigned to a “high alexithymia” group (*n* = 30) and those with scores below the cutoff to a “low alexithymia” group (*n* = 107). The average IA score was .49 (*SD* = .28) among the low alexithymia group and .55 (*SD* = .26) among the high alexithymia group, a difference that was small and statistically nonsignificant, *t*(135) = 1.04, *p* = .30, *d* = 0.22, BF_10_ = 0.35. The mean IA score for each time interval on the heartbeat detection task among each alexithymia group is presented in Table 2. A 2 (group: high alexithymia/low alexithymia) × 4 (time interval: 25 s/35 s/45 s/100 s) analysis of variance (ANOVA) was conducted on this data. Results revealed nonsignificant main effects of time interval, *F*(3, 405) = 1.13, *p* = .34, η_*p*_^2^ = .008, and group, *F*(1, 135) = 1.08, *p* = .30, η_*p*_^2^ = .008. Moreover, the Group × Time Interval interaction effect was also nonsignificant, *F*(3, 405) = 1.65, *p* = .18, η_*p*_^2^ = .01. Thus, there were no significant differences between the high and low alexithymia groups in terms of either overall level or patterns of IA on the heartbeat-tracking task.[Table-anchor tbl2]

#### Cross-validation of results

We assessed the reliability of the current findings by randomly splitting our sample into two groups of *n* = 68 and 69 participants, respectively, and reanalyzing the data in each subsample. The results are presented in full in Supplement 1. In summary, results were identical in each subsample and replicated the results observed in the full sample of 137.

## Experiment 2

### Method

#### Participants

Forty-six adults with ASD and 48 NT comparison adults aged between 20 and 64 years were recruited and tested either at City, University of London, or the University of Kent. Verbal, performance, and full-scale IQ (VIQ, PIQ, and FSIQ, respectively) scores were gained for all participants using either the Wechsler Abbreviated Scale for Intelligence-II (Wechsler, 1999; *n* = 38), or the Wechsler Adult Scales of Intelligence (Wechsler, 2008; *n* = 56). Participants in the ASD group had received verified diagnoses, according to conventional criteria (APA, 2000; World Health Organization, 1993). In addition, participants with ASD completed the Autism Diagnostic Observation Schedule (ADOS; Lord et al., 2000), a detailed observational assessment of ASD features. Participant groups were closely matched for age, sex, VIQ, PIQ, and FSIQ, but differed significantly in AQ, TAS, and RMIE scores (Table 3). All participants in the ASD group scored above the ASD cutoff score of 7 on the ADOS and/or 26 on the AQ. Ten of the 46 participants with ASD scored above the ASD cutoff on the AQ only (with ADOS scores of 6, 6, 6, 6, 5, 5, 5, 4, 3, 3, respectively). Importantly, none of the results reported in Experiment 2 changed substantively when these 10 participants with ASD were excluded from analyses (see the online supplemental materials).[Table-anchor tbl3]

#### Materials and procedures

Participants from each group completed a version of the heartbeat-tracking task used in Experiment 1. Thirty-eight (17 ASD, 21 NT) completed the heartbeat-tracking task at Location 1 (University of Kent), using identical materials and procedures as in Experiment 1. The remaining 56 participants completed the heartbeat-tracking task at Location 2 (City, University of London), using almost identical materials and procedures as in Experiment 1. The only difference was that, rather than sitting with their eyes closed and an auditory tone signaling the start and stop of each interval, participants saw a start/stop signal on a screen (a heart appeared along with the word “Go” at the start, then the heart disappeared at the end and was replaced by “Stop”) followed by a screen that asked participants to enter their count using the keyboard. In addition, the hardware used to record heartbeats was different and consisted of an ADInstruments PowerLab unit (ML845) with a bioelectrical signal amplifier (ML408) that recorded the ECG signal through three shielded snap-connect electrodes placed on the participants chest and elbow (the reference electrode). LabChart 7 (ADInstruments, 1994–2004) software was used to record the raw ECG signal at 1 kHz and the data were processed offline to extract the heartbeat frequencies. A “Stop” and “Go” signal was superimposed on the ECG trace through a hardware link with the stimulus presentation computer. Importantly, there were no systematic differences in results across the two locations.

#### Statistical power and analysis

Given ambiguities in the methods and results of previous studies, we based our sample size on that required to detect a small-to-moderate overall group difference in IA, given that it is arguable that any effect smaller than this is unlikely to be clinically significant (even if it is statistically significant). A sample of 94 participants provides sufficient power to detect a between-groups difference of 0.50 if it exists. Crucially, Bayesian analyses were used to supplement null hypothesis significance testing.

### Results

**Figure 1 fig1:**
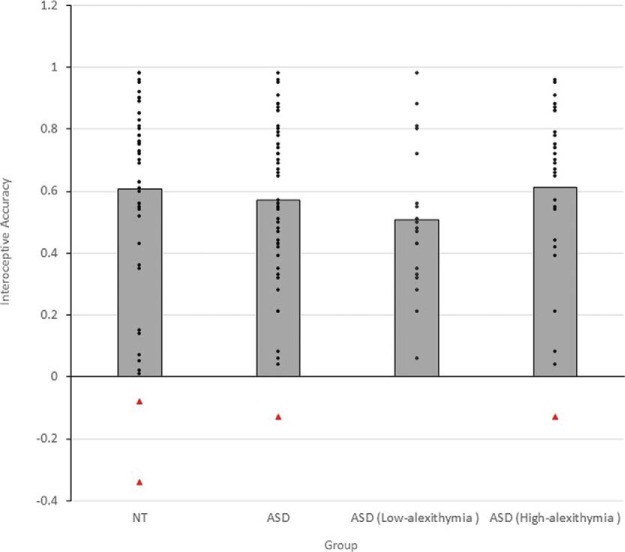
Mean interoceptive accuracy (IA) across all time intervals, among the autism spectrum disorder (ASD) group (*n* = 46), NT group (*n* = 48), ASD group with low-alexithymia (*n* = 19), and ASD group with high alexithymia *n* = (27), reported in Experiment 2. Individual data points have been indicated using black dots and outliers (defined as IA scores ±2 *SD* above/below the group mean) are indicated using red triangles. See the online article for the color version of this figure.

The average IA score was .57 (*SD* = .27) among the ASD group and .61 (*SD* = .32) among the NT group, a difference that was statistically small and nonsignificant, *t*(92) = 0.63, *p* = .53, *d* = 0.13, BF_10_ = 0.26. The mean IA score for each time interval on the heartbeat detection task in each diagnostic group is shown in Figure 1. A 2 (group: ASD/NT) × 4 (time interval: 25s/35s/45s/100s) ANOVA was conducted on this data. Neither the main effect of group, *F*(1, 92) = 0.39, *p* = .53, η_*p*_^2^ = .004, nor the Group × Time Interval interaction effect, *F*(3, 405) = 1.65, *p* = .18, η_*p*_^2^ = .01, was significant. Thus, there were no significant differences between the ASD and comparison groups in terms of either overall level of IA or patterns of IA across the four time intervals.[Fig-anchor fig1]

Out of 46 participants with ASD, 27 (58.7%) scored over the TAS cutoff for alexithymia, compared with only three of 48 (6.3%) of NT participants, χ^2^ = 29.73, *p* < .001, φ = .56, which is comparable to the prevalence estimates of clinically significant alexithymia in ASD as estimated in the sample of (e.g., Hill et al., 2004). Although the analyses above indicated that the ASD group *as a whole* did not manifest a deficit in interoception, it is possible that (in accordance with the alexithymia hypothesis) IA would be diminished among those participants with ASD who self-report clinically significant levels of alexithymia on the TAS. In order to investigate this, the ASD sample was divided according to TAS score. Those with scores above the cutoff were assigned to a high alexithymia ASD group and those with scores below the cutoff to a low alexithymia ASD group. These subsamples were matched in terms of age, VIQ, PIQ, FSIQ, sex, ADOS total score, and RMIE total score, all *p*s > .40, all *d*s < 0.27.

The average IA score on the heartbeat detection task was .51 (*SD* = .24) among the low alexithymia subsample and .61 (*SD* = .29) among the high alexithymia subsample, a difference that was small and statistically nonsignificant, *t* = 1.30, *p* = .20, *d* = 0.39, BF_10_ = 0.58. The mean IA score for each time interval on the heartbeat detection task among each subsample of ASD participants is shown in Figure 1. A 2 (subsample: high alexithymia/low alexithymia) × 4 (time interval: 25 s/35 s/45 s/100 s) ANOVA was conducted on this data. Neither the main effect of subsample, *F*(1, 44) = 1.69, *p* = .20, η_*p*_^2^ = .04, nor the Subsample × Time Interval interaction effect, *F*(3, 132) = 0.57, *p* = .64, η_*p*_^2^ = .01, was significant. Thus, there were no significant differences between the high and low alexithymia subsamples of ASD participants in terms of either overall level or patterns of IA.

In terms of associations, IA was nonsignificantly associated with TAS total score among both participants with ASD, *r* = .08, *p* = .59, BF_10_ = 0.21, and NT participants, *r* = .21, *p* = .16, BF_10_ = 0.47. Likewise, IA was nonsignificantly associated with AQ total score among both participants with ASD, *r* = .03, *p* = .83, BF_10_ = 0.19, and NT participants, *r* = .21, *p* = .16, BF_10_ = 0.47. Fisher’s *Z* tests revealed that the IA × TAS total score correlation was significantly smaller in magnitude than those reported by Herbert et al. (2011) and Shah, Hall, et al. (2016) among both participants with ASD (all *Z*s > 2.00, all *p*s < .04) and NT participants (all *Z*s > 2.00, all *p*s < .001).^3^

## General Discussion

The current findings appear to provide a significant challenge to recent theories of the mechanisms underlying the ASD phenotype in general, as well to theories of self-awareness in this disorder. In terms of theories of the phenotype, within a predictive coding framework, it has been suggested that interoceptive inference might be impaired in ASD due to a developmental pathophysiology related to oxytocin (Quattrocki & Friston, 2014). Yet, in our experiments, we found no evidence that interoception was associated with autistic traits or that it was impaired in adults with ASD, despite the fact that the ASD adults in Experiment 2 displayed the characteristic impairments in attributing mental/emotional states to others (on the RMIE task) that are argued to be one of the consequences of interoceptive impairments (see Table 3). The significant between-groups difference in performance on the RMIE task was large and associated with a Bayes factor that strongly suggested the ASD group had a mind reading impairment. In contrast, the group difference in accuracy on the heartbeat-tracking task was nonsignificant, and associated with only a negligible effect size and a Bayes factor that supported the null hypothesis. Moreover, there was no significant association between RMIE and IA in any of the samples we tested in either experiment, which again counterindicates the claim of predictive coding theories that interoception contributes significantly to social-cognitive abilities (in addition to underpinning the behavioral impairments diagnostic of ASD).

One point to note here is that we did not collect any index of participants’ body mass index (BMI), or levels of mental health difficulties. High BMI values can result in attenuated IA (e.g., Herbert et al., 2011), as can high levels of depression and anxiety (Garfinkel, Tiley, et al., 2016; but see Palser et al., 2018). The literature on BMI in adults with ASD is not entirely consistent, but shows a clear trend for adults with this condition to be overweight/obese, on average (Eaves & Ho, 2008; Jones et al., 2016; Ogden, Carroll, Kit, & Flegal, 2014; Tyler, Schramm, Karafa, Tang, & Jain, 2011). If our sample was representative, we might assume that there was a greater proportion of overweight individuals with ASD than overweight NT individuals. In that case, the participants with ASD would have been at a disadvantage on the heartbeat detection task. Likewise, rates of depression and anxiety are significantly higher among people with ASD (∼44%; Simonoff et al., 2008) than among people in the general population (∼9%; McManus, Bebbington, Jenkins, & Brugha, 2016), so participants with ASD might have been at a disadvantage on the heartbeat-tracking task. It seems highly unlikely that both the ASD and control samples were so unrepresentative that rates of depression and anxiety were greater in control participants than in ASD participants. If this was the case (and if controls had elevated rates of undiagnosed depression and anxiety), then several other results should have been apparent, but they were not. For example, there is evidence that mind reading, particularly for emotional states like those involved in the RMIE task, is diminished in people with depression/anxiety (e.g., Bourke, Douglas, & Porter, 2010; Ferguson & Cane, 2017; Wang, Wang, Chen, Zhu, & Wang, 2008; Wolkenstein, Schönenberg, Schirm, & Hautzinger, 2011). Likewise, depressive symptoms are associated with elevated rates of alexithymia (e.g., Herbert et al., 2011; Lyvers, Kohlsdorf, Edwards, & Thorberg, 2017). If the controls in our Experiment 2 had unusually high rates of mood disorder and if this explained the failure to find group differences in IA, then we should also have failed to observe between-groups differences in performance on the RMIE task (cf., Lee, Harkness, Sabbagh, & Jacobson, 2005) or TAS. Yet, we observed significant (and large) between-groups differences in each of these measures, as predicted and would be expected on the basis of the extensive literature on these abilities in ASD. These findings provide reassurance that we have not committed a Type II error in concluding that IA is undiminished in ASD. Moreover, the sample size in our Experiment 2 (*n* = 94) was nearly 2.5 times that of the sample size in any other study of IA in ASD. Thus, confidence in the reliability of the current findings should be relatively high. In the context of an interoceptive inference account of ASD, our findings would suggest that any potential dysregulation of the interoceptive system lies not at the level of perception, but rather interpretation and integration, and is unlikely to be specific to interoception alone.

Of course, the current results should be interpreted within the context of the heartbeat-tracking task we employed as the measure of interoception. It is possible that different results would be observed with a different measure of interoception, such as reporting changes in heart rate following administration of a beta-adrenergic agonist, which raises heart rate and blood pressure, allowing easier detection of heartbeats (Khalsa, Rudrauf, Sandesara, Olshansky, & Tranel, 2009), or reporting of respiratory or gastric interoception (Garfinkel, Manassei, et al., 2016; van Dyck et al., 2016). The heartbeat-tracking task has recently come under scrutiny over concerns that it may not always/necessarily provide a valid measure of interoception (e.g., Khalsa et al., 2009; see also Brener & Ring, 2016, for a review). While use of alternative, complementary measures will be important in future studies to develop a comprehensive view of the functional integrity of interoception in disorders such as autism and alexithymia, the predictions of the theories tested in the current study were nevertheless derived almost exclusively from studies of the heartbeat-tracking task. Almost all existing studies of a) interoception in ASD and b) the relation between interoception and alexithymia have employed this task as an objective (rather than self-report) index of IA (Garfinkel et al., 2015; Herbert et al., 2011; Schauder et al., 2015; Shah et al., 2016, 2017; but, for exceptions, see Gaigg et al., 2016; Murphy, Catmur, & Bird, 2018). Therefore, the current findings are highly relevant in that they speak to, and challenge, current thinking about what role interoceptive difficulties might play in the etiology of autism and alexithymia.

In particular, the current findings challenge previous research showing an association between alexithymia and IA. Across the two experiments reported here, we failed to find a significant association between heartbeat tracking accuracy and TAS score in any of five analyses in four entirely independent subsamples, each of which had more than sufficient statistical power to detect the predicted association. In every case, Bayesian analyses indicated that the data were in keeping with the null hypothesis. Also, in all but one analysis, Fisher’s *Z* tests indicated that the associations observed in the current study were significantly different (smaller/less negative) than those reported previously. Given the well-established “file-drawer” problem and evidence that some results in psychology are difficult to replicate (e.g., Pashler & Wagenmakers, 2012), we suggest the current results are important and should contribute to theory building in this area. For instance, it is interesting to note that a number of studies have recently reported associations between alexithymia and interoception using self-report measures of interoception (Betka et al., 2018; Brewer, Cook, & Bird, 2016; Longarzo et al., 2015). As Garfinkel et al., (2015) have recently pointed out, self-report questionnaires and objective measures of interoception such as the heartbeat-tracking task, capture dissociable aspects of interoception. Specifically, whereas self-report questionnaires provide insight into people’s *interoceptive sensibility* (i.e., the extent to which they tend to be aware of interoceptive signals), heartbeat-tracking tasks provide insight into their IA (i.e., the extent to which they perceive interoceptive signals accurately), and both dimensions can vary independently. The findings reported in the current paper suggest that IA is neither related to alexithymia nor impaired in ASD, but do not speak to the role of other facets of interoception in the etiology of either alexithymia or ASD.

Another implication of the current findings is that the results appear problematic for the alexithymia hypothesis of autism, which argues that some of the cognitive/emotional-processing difficulties which are common in the condition, are due to alexithymia and should not be characterized as central components of the ASD phenotype (Bird & Cook, 2013). The current results test several of the predictions arising from this theory. On the one hand, in Experiment 1, we found that performance on the RMIE task was associated significantly with performance on the TAS, but not the AQ (and that the TAS × RMIE correlation remained significant after controlling for AQ score). This replicates Oakley et al.’s (2016) findings and suggests, in accordance with the alexithymia hypothesis, that emotional aspects of mind reading are related to level of alexithymia rather than number of ASD traits. On the other hand, in Experiment 2, there were no significant differences between ASD participants with and without clinically significant levels of alexithymia in RMIE task performance. Thus, difficulties with emotional aspects of mind reading are still apparent in individuals with ASD who do not have alexithymia, contrary to the alexithymia hypothesis. Regardless, the assessment of the relation between alexithymia and mindreading was a secondary aim of the current study. The primary aim was to assess a different prediction stemming from the alexithymia hypothesis, namely, that only individuals with ASD who manifest high levels of alexithymia should display diminished interoception, whereas IA should be unimpaired in those with ASD who manifest low levels of self-reported alexithymia (Gaigg et al., 2016; Shah, Catmur, et al., 2016). The current study provided a complete test of this hypothesis *for the first time*. Previous studies aiming to test the alexithymia hypothesis have matched ASD and comparison groups for (subclinical) levels of alexithymia, found no between-groups differences in IA, and then claimed that *if* the participant groups had not been matched for level of alexithymia then between-groups differences in IA would have been found. But to prove such a claim, the IA of an ASD-high alexithymia group and a matched ASD-low alexithymia group needed to be compared directly. The fact that we observed no significant difference in IA between these two ASD groups (in fact there was a slight, nonsignificant trend for the ASD-high alexithymia group to show *superior* accuracy) provides a clear challenge to the alexithymia hypothesis of ASD, at least with regard to predictions about IA in heartbeat detection.

Instead, the results are in keeping with theories of self-awareness in ASD that draw a distinction between psychological and physical aspects of self. In the literature on the typical development of self-awareness, a distinction between physical and psychological aspects of self is frequently drawn (Gillihan & Farah, 2005). Several researchers have applied this framework to ASD and suggested that people with this disorder tend to have a diminished awareness of the psychological self (one’s own mental states, personality traits, autobiographical memories etc.), leaving awareness of the physical self (e.g., awareness of one’s physical appearance, physiological states, motor routines etc.) relatively undiminished (e.g., Lind, 2010; Uddin, 2011; Williams, 2010). It is important to note that these theories do not predict that people with ASD necessarily *interpret* their physiological states accurately, but merely that they can detect them accurately. Interpreting physiological states arguably requires the kind of metacognitive monitoring ability that is known to be impaired in people with this disorder (e.g., (Grainger, Williams, & Lind, 2014; Williams, Bergström, & Grainger, 2018; Williams, Lind, & Happé, 2009), whereas mere detection should not (Carruthers, 2009). At the very least, the finding of unimpaired IA among individuals with ASD in the current study provides support for this prediction that basic representation of bodily states is undiminished in ASD.

## Supplementary Material

10.1037/abn0000370.supp

## Figures and Tables

**Table 1 tbl1:** Correlations Between Interoceptive Accuracy (IA), Alexithymia (TAS-20), Autistic Traits (AQ), and Theory of Mind (RMIE) for Experiment 1 and the ASD and NT Participant Groups in Experiment 2

	Exp. 1 (*n* = 137)			Exp. 2 ASD (*n* = 46)			Exp. 2 TD (*n* = 48)		
Measure	IA	TAS-20	AQ	IA	TAS-20	AQ	IA	TAS-20	AQ
TAS-20	.008			.08			.21		
AQ	−.011	.42**		−.03	.61**		.21	.42**	
RMIE	.03	−.24*	.11	.34	−.10	−.13	.09	−.29	−.20
*Note.* Pearson’s *R* coefficients are shown, with significant associations highlighted with asterisks. IA = interoceptive accuracy; TAS-20 = Toronto Alexithymia Scale; AQ = Autism-Spectrum Quotient; RMIE = Reading the Mind in the Eyes task; ASD = autism spectrum disorder.									
* *p* < .05. ** *p* < .01.									

**Table 2 tbl2:** Interoceptive Accuracy in Experiments 1 as a Function of the Interval Duration Over Which Participants Tracked Their Heartbeat. Descriptive Statistics are Shown for Overall Group Means as Well as High and Low Alexithymia Subgroups Where Relevant

Group	25 s		35 s		45 s		100 s		Overall	
	*M*	*SD*	*M*	*SD*	*M*	*SD*	*M*	*SD*	*M*	*SD*
Experiment 1										
High alex (*n* = 30)	.55	.32	.54	.32	.58	.25	.52	.27	.55	.26
Low alex (*n* = 107)	.47	.32	.54	.32	.47	.31	.48	.33	.49	.28
Overall (*n* = 137)	.49	.32	.54	.31	.5	.3	.49	.32	.50	.27
Experiment 2										
NT (*n* = 48)	.61	.36	.62	.33	.58	.32	.61	.32	.61	.32
ASD (*n* = 46)	.61	.25	.58	.32	.56	.30	.52	.34	.57	.27
High alex ASD (*n* = 27)	.63	.29	.64	.34	.61	.32	.57	.34	.61	.29
Low alex ASD (*n* = 19)	.58	.19	.51	.27	.50	.27	.45	.34	.51	.24
*Note.* alex = alexithymia; NT = neurotypical; ASD = autism spectrum disorder.										

**Table 3 tbl3:** Experiment 2 Participant Characteristics and Matching Statistics

Measure	ASD (*n* = 46)	Comparison (*n* = 48)	*t*	*p*	*d*	BF_10_
Age	40.16 (11.72)	41.19 (12.57)	.41	.68	.09	.23
VIQ	109.98 (16.94)	111.17 (13.51)	.38	.71	.08	.23
PIQ	105.52 (17.46)	105.90 (12.67)	.11	.91	.03	.22
FSIQ	108.17 (16.91)	109.10 (12.18)	.31	.76	.06	.23
TAS	59.33 (14.17)	44.88 (9.79)	5.77	<.001	1.19	>100
AQ^a^	32.56 (7.79)	16.91 (5.64)	10.99	<.001	2.31	>100
ADOS^b^	9.40 (4.16)					
RMIE^c^	23.33 (6.17)	26.87 (3.75)	3.28	.001	.70	21.35
*Note.* ASD = autism spectrum disorder; BF_10_ = Bayes factor; VIQ = verbal IQ; PIQ = performance IQ; FSIQ = full-scale IQ; TAS = Toronto Alexithymia Scale; AQ = Autism-Spectrum Quotient; ADOS = Autism Diagnostic Observation Schedule; RMIE = Reading the Mind in the Eyes task.						
^a^ AQ data is missing for one ASD and one comparison participant. ^b^ ADOS was completed by 40/46 participants with ASD (6 participants refused to complete the task or were unable to complete it during the study). ^c^ RMIE was completed by 42/46 participants with ASD and 46/48 neurotypical participants.						

## Footnotes

1 Shah and colleagues (2016) used a slightly different calculation to measure interoceptive accuracy (IA) on the heartbeat detection task: [1 – (recorded number of heartbeats – counted number of heartbeats/recorded heartbeats)] × 100. This calculation, which produces significantly higher mean levels of IA to the calculation employed by Garfinkel, Tiley, et al. (2015), has the limitation of being open to potential positive accuracy biases in IA when participants display high variance in their responses, especially in cases where participants overestimate the number of heartbeats in a given time interval. However, for completeness we also analyzed data from Experiments 1 and 2 using the Shah calculation. Results were substantively identical using both methods. The correlation between IA calculated using the Shandry equation and IA calculated using the Garfinkel equation is almost perfect in our Experiments 1 and 2 (*r*s ≥ .98, *p*s < .001). All significant findings using the Garfinkel, Tiley, et al. (2016) scoring were significant using the Shah et al. scoring, and all nonsignificant findings using the Garfinkel et al. scoring were nonsignificant using the Shah et al. scoring.

2 To confirm that there were no systematic differences between males and females in terms of interoceptive accuracy (IA) on the heartbeat detection task, a 2 (sex: male/female) × 4 (time interval: 25 s/35 s/45 s/100 s) interaction was conducted on this data. Results revealed a nonsignificant main effect of group, *F*(1, 135) = 0.22, p = .64, η_*p*_^2^ = .002, as well as a nonsignificant Sex × Time Interval interaction, *F*(3, 405) = 0.63, *p* = .60, η_*p*_^2^ = .005. Thus, there were no significant differences between males and females in terms of either overall level of IA or patterns of accuracy across time intervals. Likewise, when correlation analyses were conducted among males and females separately, exactly the same pattern held in both sexes: IA was nonsignificantly associated with any other variables in either sex, all *r*s < .13, all *p*s > .25. Likewise, AQ and RMIE were nonsignificantly associated among both males and females, all *r*s < .15, all *p*s > .22. TAS total score was associated with both AQ total score and RMIE total among both males and females, all *r*s > .19, all *p*s < .04.

3 To confirm that there were no systematic differences in results across locations, an extra location variable was included in a second ANOVA. Hence, a 2 (location: Kent/City) × 2 (group: ASD/NT) × 4 (time interval: 25 s/35 s/45 s/100 s) interaction was conducted on interoceptive accuracy (IA) data. Main effects and interactions involving group and time interval were the same as in the original ANOVA and, most important, none of the main effects (all *p*s > .18, all η_*p*_^2^ < .02) or interaction effects (all *p*s > .28, all η_*p*_^2^ < .02) involving location were significant or greater than small in magnitude. Mean IA among participants at Kent was .66 (*SD* = .22) and was .69 (*SD* = .17) among participants at City, a difference that is small and nonsignificant, *t* = 0.83, *p* = .41, *d* = 0.15. Likewise, when correlation analyses were conducted among participants from each location separately, exactly the same pattern held in both: IA was nonsignificantly associated with any other variables in either Location 1 or Location 2, all *r*s < .12, all *p*s > .47. TAS total score was associated significantly with AQ total score at both locations, all *r*s > .68, all *p*s < .001.

## References

[c71] ADInstruments (2004). LabChart 7 (v.7.3.1.) [Computer software]. Retrieved from www.adiinstruments.com. (Original worked published 1994)

[c1] American Psychiatric Association (2000). Diagnostic and statistical manual of mental disorders (4th ed., text rev.). Washington, DC: Author.

[c2] American Psychiatric Association (2013). Diagnostic and statistical manual of mental disorders (5th ed.). Washington, DC: Author.

[c3] Bagby R. M., Parker J. D., & Taylor G. J. (1994). The twenty-item Toronto Alexithymia Scale—I. Item selection and cross-validation of the factor structure. Journal of Psychosomatic Research, 38, 23–32. 10.1016/0022-3999(94)90005-1 8126686

[c4] Baron-Cohen S., Wheelwright S., Hill J., Raste Y., & Plumb I. (2001). The “Reading the Mind in the Eyes” Test revised version: A study with normal adults, and adults with Asperger syndrome or high-functioning autism. Journal of Child Psychology and Psychiatry, 42, 241–251. 10.1111/1469-7610.00715 11280420

[c5] Baron-Cohen S., Wheelwright S., Skinner R., Martin J., & Clubley E. (2001). The Autism-Spectrum Quotient (AQ): Evidence from Asperger syndrome/high-functioning autism, males and females, scientists and mathematicians. Journal of Autism and Developmental Disorders, 31, 5–17. 10.1023/A:1005653411471 11439754

[c6] Barrett L. F., Quigley K. S., Bliss-Moreau E., & Aronson K. R. (2004). Interoceptive sensitivity and self-reports of emotional experience. Journal of Personality and Social Psychology, 87, 684–697. 10.1037/0022-3514.87.5.684 15535779 PMC1224728

[c7] Berthoz S., & Hill E. L. (2005). The validity of using self-reports to assess emotion regulation abilities in adults with autism spectrum disorder. European Psychiatry, 20, 291–298. 10.1016/j.eurpsy.2004.06.013 15935431

[c8] Betka S., Pfeifer G., Garfinkel S., Prins H., Bond R., Sequeira H., . . . Critchley H. (2018). How do self-assessment of alexithymia and sensitivity to bodily sensations relate to alcohol consumption? Alcoholism: Clinical and Experimental Research, 42, 81–88. 10.1111/acer.13542 29094768

[c9] Bird G., & Cook R. (2013). Mixed emotions: The contribution of alexithymia to the emotional symptoms of autism. Translational Psychiatry, 3, e285 10.1038/tp.2013.61 23880881 PMC3731793

[c10] Bird G., Silani G., Brindley R., White S., Frith U., & Singer T. (2010). Empathic brain responses in insula are modulated by levels of alexithymia but not autism. Brain: A Journal of Neurology, 133, 1515–1525. 10.1093/brain/awq060 20371509 PMC2859151

[c72] Bourke C., Douglas K., & Porter R. (2010). Processing of facial emotion expression in major depression: A review. Australian and New Zealand Journal of Psychiatry, 44, 681–696. 20636189 10.3109/00048674.2010.496359

[c11] Brener J., & Ring C. (2016). Towards a psychophysics of interoceptive processes: The measurement of heartbeat detection. Philosophical Transactions of the Royal Society, B, 371, 20160015 10.1098/rstb.2016.0015 PMC5062103 28080972

[c12] Brewer R., Cook R., & Bird G. (2016). Alexithymia: A general deficit of interoception. Royal Society Open Science, 3, 150664 10.1098/rsos.150664 27853532 PMC5098957

[c13] Brewer R., Happé F., Cook R., & Bird G. (2015). Commentary on “Autism, oxytocin and interoception”: Alexithymia, not autism spectrum disorders, is the consequence of interoceptive failure. Neuroscience and Biobehavioral Reviews, 56, 348–353. 10.1016/j.neubiorev.2015.07.006 26192103

[c14] Carruthers P. (2009). How we know our own minds: The relationship between mindreading and metacognition. Behavioral and Brain Sciences, 32, 121–138. 10.1017/S0140525X09000545 19386144

[c15] Cascio C. J., Foss-Feig J. H., Burnette C. P., Heacock J. L., & Cosby A. A. (2012). The rubber hand illusion in children with autism spectrum disorders: Delayed influence of combined tactile and visual input on proprioception. Autism, 16, 406–419. 10.1177/1362361311430404 22399451 PMC3529180

[c16] Christensen J. F., Gaigg S. B., & Calvo-Merino B. (2018). I can feel my heartbeat: Dancers have increased interoceptive accuracy. Psychophysiology, 55, e13008 10.1111/psyp.13008 28940488

[c17] Cook R., Brewer R., Shah P., & Bird G. (2013). Alexithymia, not autism, predicts poor recognition of emotional facial expressions. Psychological Science, 24, 723–732. 10.1177/0956797612463582 23528789

[c18] Craig A. D. (2003). Interoception: The sense of the physiological condition of the body. Current Opinion in Neurobiology, 13, 500–505. 10.1016/S0959-4388(03)00090-4 12965300

[c74] Critchley H. D., Wiens S., Rotshtein P., Öhman A., & Dolan R. J. (2004). Neural systems supporting interoceptive awareness. Nature Neuroscience, 7, 189 10.1038/nn1176 14730305

[c19] Dawson G., & McKissick F. C. (1984). Self-recognition in autistic children. Journal of Autism and Developmental Disorders, 14, 383–394. 10.1007/BF02409829 6520093

[c20] Demers L. A., & Koven N. S. (2015). The relation of alexithymic traits to affective theory of mind. The American Journal of Psychology, 128, 31–42. 10.5406/amerjpsyc.128.1.0031 26219172

[c21] Dienes Z. (2014). Using Bayes to get the most out of non-significant results. Frontiers in Psychology, 5, 781 10.3389/fpsyg.2014.00781 25120503 PMC4114196

[c76] Dunn B. D., Galton H. C., Morgan R., Evans D., Oliver C., Meyer M., . . . Dalgleish T. (2010). Listening to your heart: How interoception shapes emotion experience and intuitive decision making. Psychological Science, 21, 1835–1844. 21106893 10.1177/0956797610389191

[c77] Eaves L. C., & Ho H. H. (2008). Young adult outcome of autism spectrum disorders. Journal of Autism and Developmental Disorders, 38, 739–747. 17764027 10.1007/s10803-007-0441-x

[c22] Faul F., Erdfelder E., Buchner A., & Lang A. G. (2009). Statistical power analyses using G*Power 3.1: Tests for correlation and regression analyses. Behavior Research Methods, 41, 1149–1160. 10.3758/BRM.41.4.1149 19897823

[c78] Ferguson H. J., & Cane J. (2017). Tracking the impact of depression in a perspective-taking task. Scientific Reports, 7, 14821 10.1038/s41598-017-13922-y 29093490 PMC5666009

[c23] Ferrari M., & Matthews W. S. (1983). Self-recognition deficits in autism: Syndrome-specific or general developmental delay? Journal of Autism and Developmental Disorders, 13, 317–324. 10.1007/BF01531569 6196346

[c24] Fukushima H., Terasawa Y., & Umeda S. (2011). Association between interoception and empathy: Evidence from heartbeat-evoked brain potential. International Journal of Psychophysiology, 79, 259–265. 10.1016/j.ijpsycho.2010.10.015 21055427

[c25] Gaigg S. B. (2012). The interplay between emotion and cognition in autism spectrum disorder: Implications for developmental theory. Frontiers in Integrative Neuroscience, 6, 113 10.3389/fnint.2012.00113 23316143 PMC3540960

[c26] Gaigg S. B., Cornell A. S., & Bird G. (2016). The psychophysiological mechanisms of alexithymia in autism spectrum disorder. Autism: An International Journal of Research and Practise, 22, 227–231. 10.1177/1362361316667062 27811193

[c27] Gallese V., & Sinigaglia C. (2011). What is so special about embodied simulation? Trends in Cognitive Sciences, 15, 512–519. 10.1016/j.tics.2011.09.003 21983148

[c28] Garfinkel S. N., Seth A. K., Barrett A. B., Suzuki K., & Critchley H. D. (2015). Knowing your own heart: Distinguishing interoceptive accuracy from interoceptive awareness. Biological Psychology, 104, 65–74. 10.1016/j.biopsycho.2014.11.004 25451381

[c73] Garfinkel S. N., Manassei M. F., Hamilton-Fletcher G., den Bosch Y. I., Critchley H. D., & Engels M. (2016). Interoceptive dimensions across cardiac and respiratory axes. Philosophical Transactions of the Royal Society B: Biological Sciences, 371, 20160014 10.1098/rstb.2016.0014 PMC5062102 28080971

[c29] Garfinkel S. N., Tiley C., O’Keeffe S., Harrison N. A., Seth A. K., & Critchley H. D. (2016). Discrepancies between dimensions of interoception in autism: Implications for emotion and anxiety. Biological Psychology, 114, 117–126. 10.1016/j.biopsycho.2015.12.003 26724504

[c30] Gillihan S. J., & Farah M. J. (2005). Is self special? A critical review of evidence from experimental psychology and cognitive neuroscience. Psychological Bulletin, 131, 76–97. 10.1037/0033-2909.131.1.76 15631554

[c31] Glenberg A. M. (2010). Embodiment as a unifying perspective for psychology. WIREs Cognitive Science, 1, 586–596. 10.1002/wcs.55 26271505

[c32] Goldman A., & de Vignemont F. (2009). Is social cognition embodied? Trends in Cognitive Sciences, 13, 154–159. 10.1016/j.tics.2009.01.007 19269881

[c33] Grainger C., Williams D. M., & Lind S. E. (2014). Metacognition, metamemory, and mindreading in high-functioning adults with autism spectrum disorder. Journal of Abnormal Psychology, 123, 650–659. 10.1037/a0036531 24955572

[c34] Hatfield T. R., Brown R. F., Giummarra M. J., & Lenggenhager B. (2017). Autism spectrum disorder and interoception: Abnormalities in global integration? Autism. Advance online publication 10.1177/1362361317738392 29139302

[c35] Herbert B. M., Herbert C., & Pollatos O. (2011). On the relationship between interoceptive awareness and alexithymia: Is interoceptive awareness related to emotional awareness? Journal of Personality, 79, 1149–1175. 10.1111/j.1467-6494.2011.00717.x 21241306

[c36] Hill E., Berthoz S., & Frith U. (2004). Brief report: Cognitive processing of own emotions in individuals with autistic spectrum disorder and in their relatives. Journal of Autism and Developmental Disorders, 34, 229–235. 10.1023/B:JADD.0000022613.41399.14 15162941

[c37] JASP Team (2016). JASP (Version 0.8.1) [Computer software]. Retrieved from https://jasp-stats.org/

[c38] Jeffreys H. (1961). Theory of probability (3rd ed.). New York, NY: Oxford University Press, Clarendon Press.

[c75] Jones K. B., Cottle K., Bakian A., Farley M., Bilder D., Coon H., & McMahon W. M. (2016). A description of medical conditions in adults with autism spectrum disorder: A follow-up of the 1980s Utah/UCLA Autism Epidemiologic Study. Autism, 20, 551–561. 26162628 10.1177/1362361315594798

[c39] Joukamaa M., Taanila A., Miettunen J., Karvonen J. T., Koskinen M., & Veijola J. (2007). Epidemiology of alexithymia among adolescents. Journal of Psychosomatic Research, 63, 373–376. 10.1016/j.jpsychores.2007.01.018 17905044

[c40] Khalsa S. S., Rudrauf D., Sandesara C., Olshansky B., & Tranel D. (2009). Bolus isoproterenol infusions provide a reliable method for assessing interoceptive awareness. International Journal of Psychophysiology, 72, 34–45. 10.1016/j.ijpsycho.2008.08.010 18854201 PMC3085829

[c79] Lee L., Harkness K. L., Sabbagh M. A., & Jacobson J. A. (2005). Mental state decoding abilities in clinical depression. Journal of Affective Disorders, 86, 247–258. 15935244 10.1016/j.jad.2005.02.007

[c42] Lind S. E. (2010). Memory and the self in autism. Autism, 14, 430–456. 10.1177/1362361309358700 20671017

[c43] Lind S. E., & Bowler D. M. (2009). Delayed self-recognition in children with autism spectrum disorder. Journal of Autism and Developmental Disorders, 39, 643–650. 10.1007/s10803-008-0670-7 19051001

[c44] Longarzo M., D’Olimpio F., Chiavazzo A., Santangelo G., Trojano L., & Grossi D. (2015). The relationships between interoception and alexithymic trait. The Self-Awareness Questionnaire in healthy subjects. Frontiers in Psychology, 6, 1149 10.3389/fpsyg.2015.01149 26300829 PMC4528101

[c45] Lord C., Risi S., Lambrecht L., Cook E. H. Jr., Leventhal B. L., DiLavore P. C., . . . Rutter M. (2000). The autism diagnostic observation schedule-generic: A standard measure of social and communication deficits associated with the spectrum of autism. Journal of Autism and Developmental Disorders, 30, 205–223. 10.1023/A:1005592401947 11055457

[c80] Lyvers M., Kohlsdorf S. M., Edwards M. S., & Thorberg F. A. (2017). Alexithymia and mood: Recognition of emotion in self and others. American Journal of Psychology, 130, 83–92. 29508959 10.5406/amerjpsyc.130.1.0083

[c81] McManus S., Bebbington P., Jenkins R., & Brugha T. (Ed.). (2016). Mental health and wellbeing in England: Adult psychiatric morbidity survey 2014. Leeds, England: NHS Digital Retrieved from https://mhfe.org.uk/content/mental-health-and-wellbeing-england-adult-psychiatric-morbidity-survey-2014

[c46] Milosavljevic B., Carter Leno V., Simonoff E., Baird G., Pickles A., Jones C. R., . . . Happé F. (2016). Alexithymia in adolescents with autism spectrum disorder: Its relationship to internalising difficulties, sensory modulation and social cognition. Journal of Autism and Developmental Disorders, 46, 1354–1367. 10.1007/s10803-015-2670-8 26659552

[c82] Murphy J., Catmur C., & Bird G. (2018). Alexithymia is associated with a multidomain, multidimensional failure of interoception: Evidence from novel tests. Journal of Experimental Psychology: General, 147, 398 10.1037/xge0000366 29154612 PMC5824617

[c47] Mussgay L., Klinkenberg N., & Rüddel H. (1999). Heart beat perception in patients with depressive, somatoform, and personality disorders. Journal of Psychophysiology, 13, 27–36. 10.1027//0269-8803.13.1.27

[c48] Oakley B. F. M., Brewer R., Bird G., & Catmur C. (2016). Theory of mind is not theory of emotion: A cautionary note on the Reading the Mind in the Eyes Test. Journal of Abnormal Psychology, 125, 818–823. 10.1037/abn0000182 27505409 PMC4976760

[c85] Ogden C. L., Carroll M. D., Kit B. K., & Flegal K. M. (2014). Prevalence of childhood and adult obesity in the United States, 2011–2012. Jama, 311, 806–814. 24570244 10.1001/jama.2014.732 PMC4770258

[c86] Palser E. R., Fotopoulou A., Pellicano E., & Kilner J. M. (2018). The link between interoceptive processing and anxiety in children diagnosed with autism spectrum disorder: Extending adult findings into a developmental sample. Biological Psychology, 136, 13–21. 29742462 10.1016/j.biopsycho.2018.05.003

[c49] Pashler H., & Wagenmakers E.-J. (2012). Editors’ introduction to the special section on replicability in psychological science: A crisis of confidence? Perspectives on Psychological Science, 7, 528–530. 10.1177/1745691612465253 26168108

[c50] Paton B., Hohwy J., & Enticott P. G. (2012). The rubber hand illusion reveals proprioceptive and sensorimotor differences in autism spectrum disorders. Journal of Autism and Developmental Disorders, 42, 1870–1883. 10.1007/s10803-011-1430-7 22189963

[c88] Pollatos O., Schandry R., Auer D. P., & Kaufmann C. (2007). Brain structures mediating cardiovascular arousal and interoceptive awareness. Brain Research, 1141, 178–187. 17296169 10.1016/j.brainres.2007.01.026

[c51] Quattrocki E., & Friston K. (2014). Autism, oxytocin and interoception. Neuroscience and Biobehavioral Reviews, 47, 410–430. 10.1016/j.neubiorev.2014.09.012 25277283 PMC4726659

[c53] Rouder J. N., Speckman P. L., Sun D., Morey R. D., & Iverson G. (2009). Bayesian t tests for accepting and rejecting the null hypothesis. Psychonomic Bulletin & Review, 16, 225–237. 10.3758/PBR.16.2.225 19293088

[c54] Schandry R. (1981). Heart beat perception and emotional experience. Psychophysiology, 18, 483–488. 10.1111/j.1469-8986.1981.tb02486.x 7267933

[c55] Schauder K. B., Mash L. E., Bryant L. K., & Cascio C. J. (2015). Interoceptive ability and body awareness in autism spectrum disorder. Journal of Experimental Child Psychology, 131, 193–200. 10.1016/j.jecp.2014.11.002 25498876 PMC4303499

[c56] Seth A. K. (2013). Interoceptive inference, emotion, and the embodied self. Trends in Cognitive Sciences, 17, 565–573. 10.1016/j.tics.2013.09.007 24126130

[c57] Shah P., Catmur C., & Bird G. (2016). Emotional decision-making in autism spectrum disorder: The roles of interoception and alexithymia. Molecular Autism, 7, 43 10.1186/s13229-016-0104-x 27777716 PMC5062918

[c58] Shah P., Catmur C., & Bird G. (2017). From heart to mind: Linking interoception, emotion, and theory of mind. Cortex: A Journal Devoted to the Study of the Nervous System and Behavior, 93, 220–223. 10.1016/j.cortex.2017.02.010 28476292 PMC5542037

[c59] Shah P., Hall R., Catmur C., & Bird G. (2016). Alexithymia, not autism, is associated with impaired interoception. Cortex: A Journal Devoted to the Study of the Nervous System and Behavior, 81, 215–220. 10.1016/j.cortex.2016.03.021 27253723 PMC4962768

[c83] Simonoff E., Pickles A., Charman T., Chandler S., Loucas T., & Baird G. (2008). Psychiatric disorders in children with autism spectrum disorders: Prevalence, comorbidity, and associated factors in a population-derived sample. Journal of the American Academy of Child & Adolescent Psychiatry, 47, 921–929. 18645422 10.1097/CHI.0b013e318179964f

[c60] Taylor G. J. (1984). Alexithymia: Concept, measurement, and implications for treatment. The American Journal of Psychiatry, 141, 725–732. 10.1176/ajp.141.6.725 6375397

[c84] Tyler C. V., Schramm S. C., Karafa M., Tang A. S., & Jain A. K. (2011). Chronic disease risks in young adults with autism spectrum disorder: Forewarned is forearmed. American Journal on Intellectual and Developmental Disabilities, 116, 371–380. 21905805 10.1352/1944-7558-116.5.371

[c61] Uddin L. Q. (2011). The self in autism: An emerging view from neuroimaging. Neurocase, 17, 201–208. 10.1080/13554794.2010.509320 21207316 PMC3117464

[c62] van Dyck Z., Vögele C., Blechert J., Lutz A. P. C., Schulz A., & Herbert B. M. (2016). The Water Load Test as a measure of gastric interoception: Development of a two-stage protocol and application to a healthy female population. PLoS ONE, 11, e0163574 10.1371/journal.pone.0163574 27657528 PMC5033375

[c87] Wang Y. G., Wang Y. Q., Chen S. L., Zhu C. Y., & Wang K. (2008). Theory of mind disability in major depression with or without psychotic symptoms: A componential view. Psychiatry Research, 161, 153–161. 18926572 10.1016/j.psychres.2007.07.018

[c63] Wechsler D. (1999). Wechsler Abbreviated Scale of Intelligence. San Antonio, TX: The Psychological Corporation.

[c64] Wechsler D. (2008). Wechsler Adult Intelligence Scale (4th ed.). San Antonio, TX: NCS Pearson.

[c65] Williams D. (2010). Theory of own mind in autism. Autism, 14, 474–494. 10.1177/1362361310366314 20926458

[c66] Williams D. M., Bergström Z., & Grainger C. (2018). Metacognitive monitoring and the hypercorrection effect in autism and the general population: Relation to autism(-like) traits and mindreading. Autism, 22, 259–270. 29671645 10.1177/1362361316680178

[c67] Williams D. M., Lind S. E., & Happé F. (2009). Metacognition may be more impaired than mindreading in autism. Behavioral and Brain Sciences, 32, 162–163. 10.1017/S0140525X09000818

[c89] Wolkenstein L., Schönenberg M., Schirm E., & Hautzinger M. (2011). I can see what you feel, but I can’t deal with it: Impaired theory of mind in depression. Journal of Affective Disorders, 132, 104–111. 21420177 10.1016/j.jad.2011.02.010

[c68] World Health Organization (1993). International classification of mental and behavioural disorders: Clinical descriptions and diagnostic guidelines (10th ed.). Geneva, Switzerland: Author.

[c69] Yirmiya N., Erel O., Shaked M., & Solomonica-Levi D. (1998). Meta-analyses comparing theory of mind abilities of individuals with autism, individuals with mental retardation, and normally developing individuals. Psychological Bulletin, 124, 283–307. 10.1037/0033-2909.124.3.283 9849110

[c70] Zaki J., Davis J. I., & Ochsner K. N. (2012). Overlapping activity in anterior insula during interoception and emotional experience. NeuroImage, 62, 493–499. 10.1016/j.neuroimage.2012.05.012 22587900 PMC6558972

