# Supplementary material for: Interoceptive Impairments Do Not Lie at the Heart of Autism or Alexithymia
Source: J Abnorm Psychol. 2018 Aug;127(6):612–22. doi: 10.1037/abn0000370 (PMC6089261; doi:10.1037/abn0000370)
Supplement: Supplementary file 1 [file ABN-2017-0405Suppl.zip › Supplementary material.docx]

**Supplementary material**

**1) Cross validation of results from Experiment 1.**

We assessed the reliability of the current findings by randomly splitting our sample into two groups and re-analysing the data in each sub-sample. The decision to take this approach was taken *a priori*. In Subsample 1 (n = 69; 15 high alexithymia) – just as in the total sample –interoceptive accuracy was non-significantly associated with TAS20 score, *r* = .06, *p* = .63, BF_10_ = 0.17, or any of the other variables, all *r*s < -.17, all *p*s > .18, all BF_10_ <0.37. Likewise, AQ score was associated significantly with TAS score, *r* = .43, *p* <.001, BF_10_ > 100. Finally, Fisher’s Z tests revealed that the interoceptive accuracy × TAS total score correlation was significantly different from those reported by Herbert et al. (2011), *Z* = 3.04, *p* < .001, and Shah et al., (2016), *Z* = 2.09, *p* = .04.

A 2 (Group: high alexithymia/low alexithymia) × 4 (Time interval: 25s/35s/45s/100s) was also conducted in Subsample 1. Results revealed a non-significant main effect of time interval, *F*(3, 201) = 0.83, *p* = .48, $\eta_{p}^{2}=$.01 and a non-significant Group × Time interval interaction *F*(3, 405) = 0.57, *p* = .63, $\eta_{p}^{2}=$.009. The main effect of group was borderline significant and moderate in size, *F*(1, 67) = 3.69, *p* = .06, $\eta_{p}^{2}=$.05. However, contrary to the alexithymia hypothesis, this effect reflected somewhat *superior* interoceptive accuracy among the high alexithymia group (*M* = .61, *SD* = .24) than the low alexithymia group (*M* = .44, *SD* = .30).

In Subsample 2 (n = 68; 15 high alexithymia) – just as in the total sample – interoceptive accuracy was non-significantly associated with TAS20 score, *r* = -.06, *p* = .63, BF_10_ = 0.17, or any of the other variables, all *r*s < -.12, all *p*s > .33, all BF_10_ <0.24. Likewise, AQ score was associated significantly with TAS score, *r* = .42, *p* <.001, BF_10_ > 100. Finally, Fisher’s Z tests revealed that the interoceptive accuracy × TAS total score correlation was significantly different from that reported by Herbert et al. (2011), *Z* = 2.22, *p* = .03, but non-significantly different from that reported by Shah et al. (2016) in their relatively small sample of 38 participants, *Z* = 1.51, *p* = .13.

A 2 (Group: high alexithymia/low alexithymia) × 4 (Time interval: 25s/35s/45s/100s) ANOVA was also conducted in subsample 2. All main effects and interaction effects were non-significant, all *p*s > .22, $\eta_{p}^{2}=$.02. Thus, there were no significant differences between the high and low alexithymia groups in terms of either overall level or patterns of interoceptive accuracy on the heartbeat tracking task.

**2) Exploring differences in alexithymia, ASD traits, and mindreading among high vs low interoceptors in Experiment 1**

In our investigation, one of our aims was to assess the hypothesis that high levels of alexithymia result in low levels of interoceptive accuracy (the alexithymia hypothesis). One approach we took to address this was to compare interoceptive accuracy among those with clinical levels of alexithymia with interoceptive accuracy among those with normal/low levels of alexithymia. However, an anonymous reviewer rightly noted that the direction of causation could be opposite to that suggested by the alexithymia hypothesis. That is, low levels of interoceptive accuracy could lead to high levels of alexithymia. The reviewer helpfully suggested that we explore the characteristics of those participants whose interoceptive accuracy was in the top quartile (n = 34) with those participants whose interoceptive accuracy was in the bottom quartile (n = 34). We did this *post hoc* and there were no significant differences between the groups in terms of mean levels of alexithymia, *t* = 0.53, *p* = .60, *d* = 0.13, BF_10_ = 0.28, or in the proportion of individuals in each group who scored above the TAS-20 cut-off for alexithymia, χ^2^ = 0.77, p = .38, φ = 0.11. Moreover, there were no differences between the top and bottom quartile groups in terms of either mindreading ability (RMIE performance: *t* = 0.27, *p* = .79, *d* = 0.07, BF_10_ = 0.26), or number of ASD traits (AQ score: *t* = 0.47, *p* = .64, *d* = 0.11, BF_10_ = 0.27). Thus, we found no evidence that low levels of interoceptive accuracy lead to elevated levels of alexithymia, ASD traits, or to diminished mindreading (although we note that the sample size is comparatively small and we warn in the main paper about the dangers of relying on small samples when investigating these issues).

**3) Effects of excluding participants with ASD who scored below the ASD cut-off score on the ADOS in Experiment 2**

One important question to answer is whether the lack (ASD/NT) group differences in Experiment 2 were the result of the inclusion of misdiagnosed cases of ASD in the ASD group? Ten participants with ASD did not score above the cut-off score on the ADOS and so, it could be argued, may not have ASD. It is important to note that we do not believe it optimal to think of ADOS as a measure that can be used to “validate” ASD diagnoses (a high score on ADOS is not a “biomarker” of ASD). In our view, the point of measures such as ADOS is to establish the severity of features/traits (to give the reader an impression of the characteristics of each group), rather than to classify participants as having or not having ASD. Indeed, even ADOS and the Autism Diagnostic Interview–often considered the “gold standard” ASD measures–do not have particularly high levels of sensitivity (Risi et al., 2006). As such, expert clinical judgement is effectively the “gold standard” and cannot be over-ruled by scores on a measurement tool. Nonetheless, it is important to consider the issue carefully, especially when reporting null effects, so we reanalysed our data after excluding the 10 participants in the ASD group who scored under the threshold of 7 on the ADOS. This resulted in groups of 36 participants with ASD and 48 comparison participants. Participant groups remained closely matched for age, sex, VIQ, PIQ and FSIQ (all ps ≥.77), but continued to differ significantly in AQ (ASD>NT), TAS (ASD>NT), and RMIE (ASD<NT) scores, all ps ≤.001. As detailed below, all experimental results were substantively identical to those in the fill sample of participants. Thus, it is highly unlikely that our observation of null effects of group in the main paper were due to the inclusion of inaccurately phenotyped individuals in our ASD group.

The average *interoceptive accuracy* score was .56 (SD = .29) among the ASD group and .61 (SD = .32) among the NT group, a difference that was statistically small and non-significant, *t* = 0.78, *p* = .44, *d* = 0.17, BF_10_ = 0.30. Next, a 2 (Group: ASD/NT) × 4 (Time interval: 25s/35s/45s/100s) ANOVA was conducted. Neither the main effect of group, *F* = 0.61, *p* = .44, $\eta_{p}^{2}=$.007, nor the Group × Time interval interaction effect, *F* = 1.33, *p* = .27, $\eta_{p}^{2}=$.02, was significant. Thus, there were no significant differences between the ASD and comparison groups in terms of either overall level of interoceptive accuracy or patterns of interoceptive accuracy across the four time intervals even when only participants with ASD who scored above cut-off on ADOS were included.

Next, we broke down the ASD group into those who scored above the TAS-20 cut-off for alexithymia (n = 20) and those who scored below threshold (n = 16). These sub-samples remained matched in terms of age, VIQ, PIQ, FSIQ, sex, ADOS total score, and RMIE total score, all *p*s >.32, all *d*s <0.35. The average interoceptive accuracy score on the heartbeat detection task was .50 (SD = .23) among the low alexithymia sub-sample and .60 (SD = .32) among the high alexithymia sub-sample, a difference that was small and statistically non-significant, *t* = 1.06, *p* = .30, *d* = 0.36. Next, a 2 (Subsample: high alexithymia/low alexithymia) × 4 (Time interval: 25s/35s/45s/100s) ANOVA was conducted on this data. Neither the main effect of subsample, *F* = 1.12, *p* = .30, $\eta_{p}^{2}=$.03, nor the Subsample × Time interval interaction effect, *F* = 0.19, *p* = .90, $\eta_{p}^{2}=$.006, was significant. Thus, there were no significant differences between the high and low alexithymia sub-samples of ASD participants in terms of either overall level or patterns of interoceptive accuracy.

In terms of associations, interoceptive accuracy was non-significantly associated with TAS total score among this sample of ASD participants who all met threshold on the ADOS, *r* = -.03, *p* = .88. Likewise, interoceptive accuracy was non-significantly associated with AQ, *r* = - .03, *p* = .88.

**4) Were participants using alternative strategies that allowed them to perform well despite limited interoceptive ability?**

There is evidence suggesting that a natural heart rate close to 60 beats per minute (bpm) can confer an advantage on heart beat tracking accuracy due to familiarity with the 60 second/minute rhythm of common clocks (see Knapp-Kline & Kline, 2005). That is, participants with a natural heart rate of around 60 bpm could show apparently high levels of interoceptive accuracy on the heartbeat detection task merely by silently counting seconds, rather than by monitoring their own actual heartbeats. To investigate this in the current study, we calculated the number of bpm that each participant’s heart rate deviated from 60. If the lack of between-group differences in interoceptive accuracy in the current study were because participants with ASD were using a compensatory counting strategy, rather than monitoring their own heart beats (as NT participants were), then the correlation between this “heart rate difference score” and interoceptive accuracy should be significantly larger in the ASD group than the NT group. However, this was not the case. In Exp. 2, the mean heart rate difference score was 15.03 (SD = 10.85) among participants with ASD and 14.05 (SD = 11.19) among comparison participants, a between-group difference that was small and non-significant, t = 0.43, p = .67, BF_10_ = 0.24. The association between the heart rate difference score and interoceptive accuracy was small and non-significant among both participants with ASD (*r* = -.19, *p* = .19, BF_10_ = 0.43) and comparison participants (*r* = -.20, *p* = .17, BF_10_ = 0.44). Importantly, the size of the heart rate difference score × interoceptive accuracy correlation was not significantly different across groups, Z = 0.05, p = .96. When the heart rate difference score was entered as a covariate in the ANOVAs in Experiment 2, none of the results changed substantively (once the heart rate difference score was included as a covariate, no previously non-significant result became significant and no previously significant results became non-significant). Likewise, when heart rate difference score was controlled in partial correlations, none of the results changed substantively in *either* Experiment 1 or Experiment 2 (once heart rate difference score was controlled, no previously significant correlation became non-significant and no previously non-significant correlation became significant).
